# MicroRNA-132 Interact with p250GAP/Cdc42 Pathway in the Hippocampal Neuronal Culture Model of Acquired Epilepsy and Associated with Epileptogenesis Process

**DOI:** 10.1155/2016/5108489

**Published:** 2016-08-08

**Authors:** Jinxian Yuan, Hao Huang, Xin Zhou, Xi Liu, Shu Ou, Tao Xu, Ruohan Li, Limin Ma, Yangmei Chen

**Affiliations:** ^1^Department of Neurology, Second Affiliated Hospital of Chongqing Medical University, Chongqing 400016, China; ^2^Chongqing Key Laboratory of Biochemistry and Molecular Pharmacology, Chongqing Medical University, Chongqing 400016, China

## Abstract

Increasing evidence suggests that epilepsy is the result of synaptic reorganization and pathological excitatory loop formation in the central nervous system; however, the mechanisms that regulate this process are not well understood. We proposed that microRNA-132 (miR-132) and p250GAP might play important roles in this process by activating the downstream Rho GTPase family. We tested this hypothesis using a magnesium-free medium-induced epileptic model of cultured hippocampal neurons. We investigated whether miR-132 regulates GTPase activity through p250GAP and found that Cdc42 was significantly activated in our experimental model. Silencing miR-132 inhibited the electrical excitability level of cultured epileptic neurons, whereas silencing p250GAP had an opposite effect. In addition, we verified the effect of miR-132* in vivo* and found that silencing miR-132 inhibited the aberrant formation of dendritic spines and chronic spontaneous seizure in a lithium-pilocarpine-induced epileptic mouse model. Finally, we confirmed that silencing miR-132 has a neuroprotective effect on cultured epileptic neurons; however, this effect did not occur through the p250GAP pathway. Generally, silencing miR-132 may suppress spontaneous seizure activity through the miR-132/p250GAP/Cdc42 pathway by regulating the morphology and electrophysiology of dendritic spines; therefore, miR-132 may serve as a potential target for the development of antiepileptic drugs.

## 1. Introduction

Epilepsy is a neurological disorder that is characterized by recurrent seizures that result from abnormal and synchronous firing of neurons in the brain. Approximately one-third of the patients with epilepsy do not respond to drugs and are said to have intractable epilepsy. Although the precise mechanism of seizure recurrence remains elusive, elucidation of the mechanisms involved in the transformation of a normal brain into one capable of producing recurrent seizures and of maintaining an epileptic state is essential for understanding epileptogenesis and for developing new treatments for epilepsy.

MicroRNAs, as posttranscriptional regulators for up to 60% of proteins, are a major determinant of protein levels in cells [1]. MicroRNA-132 (miR-132) is significantly upregulated during active synaptogenesis and plays important roles in spine formation and maturation [2–5]. miR-132 also regulates the inflammatory response and neuronal apoptosis after acute brain injury [6–8]. Several studies have shown that miR-132 is persistently upregulated during epileptogenesis after acute brain injury [9–13]. Because synaptic dysfunction and reorganization are the most important histopathological changes in epileptic foci [14], we aimed to investigate whether miR-132 plays a role in epileptogenesis by regulating synaptic reorganization.

p250GAP is a target of miR-132 and is enriched in the NMDA receptor complex of neuronal synapses [2]. p250GAP is a Rho family GTPase-activating protein that can interact with a variety of synaptic proteins by inhibiting the activity of downstream Rho family GTPases, including RhoA, Rac1, and Cdc42 [2, 15, 16]. It is an important cytoskeletal regulator that is regulated by neuronal activity-related signaling pathways that result in the depolymerization of the cytoskeleton and a reduction in the density and volume of dendritic spines. In the central nervous system (CNS), p250GAP has been reported to mainly regulate the activity of Rac1 and Cdc42. This study aimed to explore the possible molecular mechanisms of miR-132 and its target, p250GAP, during epileptogenesis. We also aimed to determine how GTPases are regulated by p250GAP in the pathological process of epilepsy.

## 2. Materials and Methods

### 2.1. Animals

Adult male (8–12 weeks) C57BL/6 mice were used in this study. The mice were kept in an animal room at a constant temperature (22 ± 1°C) and a 12-h light/dark cycle with free access to food and water. All experimental procedures were performed in accordance with the international guidelines for the use of animals and the guidelines of the Animal Care Committee of Chongqing Medical University, China.

### 2.2. Hippocampal Neuron Culture

Hippocampal neurons from 17- to 19-day-old embryonic mice were cultured (5 × 10^5^ cells per square centimeter) on plates coated with poly-L-lysine (Catalog number P1399, Sigma, USA) as described previously [17]. The neurons were then maintained in neurobasal medium (Catalog number 21103-049, Gibco, USA) supplemented with B27 (Catalog number 17504-044, Gibco) and 0.5 mM L-glutamine (Catalog number G3126, Sigma). Approximately 1/3 to 1/2 of the culture medium was changed every 3-4 days. Ten micromolar cytosine *β*-D-arabinofuranoside (Catalog number C1768, Sigma) was added to the culture medium at 3 days* in vitro* (DIV3) to inhibit the growth of gliocytes. The cultured neurons were stained at DIV7 with a neuron-specific marker, microtubule-associated protein 2 (MAP2) (Catalog number 11267, Abcam, USA), to evaluate the purity of the cultured neurons. Only the cultured cells whose purity was higher than 98% were used for the following experiment.

### 2.3. Induction of Spontaneous Recurrent Epileptiform Discharges (SREDs) of Cultured Hippocampal Neurons

At DIV10, SREDs were induced in the neuronal cultures by exposing the neurons to magnesium-free (MGF) medium (145 mM NaCl, 10 mM HEPES, 2.5 mM KCl, 2 mM CaCl_2_, 10 mM glucose, and 0.001 mM glycine, with the pH adjusted to 7.3 with NaOH and the osmolarity adjusted to 280–320 mOsm with sucrose), for 3 h. The sham controls were treated with nonmagnesium-free medium (non-MGF), which is MGF medium supplemented with 1 mM MgCl_2_. SREDs are typically observed within 12–24 h using patch clamp recordings and can last for the life of the neurons in culture. This hippocampal neuronal culture model of status epilepticus (SE) has been well characterized as a useful* in vitro* model of refractory SE [18].

### 2.4. Cell Transfection

A miR-132 antagomir (ant-132) was used to silence the expression level of miR-132. A nontargeting scrambled sequence (Scr) was used as a control (Catalog number miR30000067-1-10, RiboBio, China). p250GAP expression was silenced using a lentivirus (LV-shp250GAP), and the same lentivirus vector expressing GFP alone (LV-GFP) was chosen as a control (Catalog number LVCON077, GeneChem, China). Neuronal transfection was conducted according to the manufacturer's instructions, and the culture medium was completely changed 10 h after transfection.

### 2.5. Patch Clamp Recordings

The membrane potentials of neurons were measured with whole-cell current-clamp recordings using a patch clamp amplifier. A cell culture dish was mounted on the stage of an inverted microscope (IX-51, Olympus, Japan), and patch pipettes were filled with an intracellular solution containing 110 mM KAsp, 30 mM KCl, 10 mM EGTA, 10 mM HEPES, 5 mM Na-ATP, 1 mM CaCl_2_, 2 mM MgCl_2_, and 10 mM TEACl, with the pH adjusted to 7.3 with CsOH and the osmolarity adjusted to 280–300 mOsm with sucrose. The experiments were performed at room temperature (22–24°C). The pipette resistance in the intracellular solution was 2–4 MΩ. The pipette resistance and capacitance were compensated electronically after the establishment of a gigaseal. After the whole-cell capacitance was compensated, recordings were made only when the series resistance was <20 MΩ. To optimize the success of recording from pyramidal neurons, phase-bright cells were selected based on both size and pyramidal soma. Cultured neurons with small dendritic arborizations, long axons, and soma diameters of 20–26 *μ*m were selected for the electrophysiological recordings to avoid space clamp artifacts. Routinely, 60–80% series resistance compensation was employed, continually monitored, and adjusted as required. Whole-cell resistance and resting membrane potential were also monitored before and during the experiments, and a cell was accepted for study only if these parameters remained stable. Whole-cell recordings were performed using an EPC-10 amplifier (HEKA, Germany) in the current-clamp mode. Data were collected and analyzed using Clamp-fit 10.0 software (Axon, USA).

### 2.6. SYBR-Green Quantitative Real-Time PCR (qRT-PCR)

Reverse transcription (RT) reactions were performed using a PrimeScript*™* RT Reagent Kit (Catalog number RR047A, TaKaRa, China). miRNA-specific stem-loop primers were used for RT of miR-132. Samples were run at 37°C for 15 min and 85°C for 5 sec, followed by a hold at 4°C. RT products were stored undiluted at −20°C prior to running real-time PCR. Real-time PCR was carried out on a Bio-Rad real-time PCR system. SYBR® Premix Ex Taq*™* II (TaKaRa, China) was used. The PCR mixture contained 12.5 *μ*L of SYBR Premix Ex Taq II, 1 *μ*L of 10 *μ*M PCR forward primer, 1 *μ*L of 10 *μ*M PCR reverse primer, 2 *μ*L of the RT reaction solution, and 8.5 *μ*L of ddH_2_O. Real-time PCR was performed under the following conditions: stage 1, 95°C for 30 s; stage 2, 40 cycles at 95°C for 5 s and 60°C for 30 s; and stage 3, dissociation. The primers used were (miR-132 RT) 5′-GTCGTATCCAGTGCAGGGTCCGAGGTATTCGCACTGGATACGACCGACCA-3′, (miR-132 forward primer) 5′-GCGGCGGTAACAGTCTACAGCC-3′, and (miR-132 reverse primer) 5′-ATCCAGTGCAGGGTCCGAGG-3′. The data were analyzed with Bio-Rad CFX Manager software; the data are represented as the mean 2^−ΔΔCT^ ± standard deviation (SD).

### 2.7. Western Blot (WB)

Total proteins were extracted using a whole protein extraction kit (Catalog number P0013, Beyotime, China). The total protein concentrations were determined using an Enhanced Bicinchoninic Acid Protein (BCA) Assay Kit (Catalog number P0012s, Beyotime, China), and the samples were stored at −20°C until use. WB analysis was performed as described previously [8]. The primary antibodies used were goat anti-p250GAP (1 : 1,000, Catalog number 138167, Santa Cruz, USA) and rabbit anti-cleaved caspase-3 (1 : 150, Catalog number #9661, CST, USA).

### 2.8. Measurements of Rac1 and Cdc42 Activation

The activation of Rac1 and Cdc42 was measured using a Rac1/Cdc42 Activation Assay Kit (Catalog number 17-441, Millipore, USA) according to the manufacturer's protocol. This assay uses the downstream effector of Rac/Cdc42, p21-activated protein kinase (PAK1), to isolate the active GTP-bound form of Rac/Cdc42 from the sample. The p21-binding domain (PBD) of PAK1 is expressed as a GST fusion protein and coupled to agarose beads. After the proteins were precipitated, an immunoblot was performed, and the activated Rac1 and Cdc42 were detected with specific monoclonal antibodies followed by an HRP-conjugated secondary antibody.

### 2.9. TdT-Mediated dUTP Nick-End Labeling (TUNEL) Assay

A TUNEL assay Roche Kit (Catalog number 11684817910, Roche, Switzerland) was used to detect the apoptosis level of cultured hippocampal neurons according to the manufacturer's instructions. Briefly, after cortical neurons were fixed in freshly prepared 4% formaldehyde solution in phosphate-buffered saline (PBS) for 20 min at room temperature and permeabilized with 0.2% Triton X-100 for 5 min, they were incubated with 50 *μ*L of a TUNEL reaction mixture for 60 min at 37°C in the dark and then rinsed with PBS (pH 7.4) 3 times for 5 min each. To detect the nuclei, the slides were incubated with DAPI for 5 min at room temperature in the dark and observed with a fluorescence microscope. The apoptotic index was expressed as the ratio of the number of TUNEL-positive neurons to the total number of neurons.

### 2.10. Temporal Lobe Epilepsy (TLE) Experimental Mouse Models and Ant-132 Intervention

The pilocarpine model has been widely used to simulate human TLE. For SE induction, all the mice were intraperitoneally (i.p.) injected with pilocarpine (300 mg/kg, 0.1 mL/10 g, i.p., Sigma). Atropine sulfate (1 mg/kg, i.p.) was administered 30 min prior to the first dose of pilocarpine. The mice that developed a stage 4 or 5 seizure (Racine's scale) [19] within 2 h after pilocarpine administration were considered kindled in our study. Diazepam (10 mg/kg, i.p.) was given to terminate the convulsions 1 h after SE onset. All of the mice were allowed to recover for 48 h after SE and were then intracerebroventricularly (i.c.v.) injected with 2 *μ*L of saline (TLE group), Scr-132 (TLE+Scr-132 group), or ant-132 (TLE+ant-132 group). The dose of ant-132 was 1 nmol, which was achieved by dilution in double-distilled water.

### 2.11. Analysis of Spontaneous Seizures by Continuous Video Monitoring

Animals in the chronic period with spontaneous recurrent seizures (SRS) associated with pilocarpine epilepsy were recorded every day for 24 h using a closed circuit video system to detect the class 4 and class 5 seizures at the 6th week. An observer who was blinded to the study reviewed the videos. Seizures were counted using a modified six-point Racine scale. Clinical events with a score below 2 were excluded.

### 2.12. Golgi-Cox Staining

A Hito Golgi-Cox OptimStain Kit (Catalog number HTKNS1125, Hitobiotec Inc., USA) was used to visualize the dendritic spines. Brain tissues obtained from all the mice in the ant-132-treated and Scr-132 control groups in our experiment were sectioned into 100-*μ*m coronal sections on a cryostat (Leica, Germany), and the staining process was conducted following the manufacturer's instructions.

### 2.13. Morphological Analysis and Density Quantification of Dendritic Spines

For morphometric measurements, at least 15 neurons were analyzed for each data point reported, and 3 × 15 *μ*m of dendrites in each neuron was chosen. The IMARIS FilamentTracer module (Andor Technology; Belfast, Northern Ireland) was used to detect, quantify, and characterize spine structures. Filopodia were defined as dendritic protrusions with mean head width ≤ mean neck width. Thin spines were defined as dendritic protrusions with two times mean neck width < spine length and with mean neck width ≤ maximum head width. Stubby spines were defined as dendritic protrusions with length < 1 *μ*m. Mushroom spines were defined as dendritic protrusions with mean head width > mean neck width. To assess the reliability of the counting of dendritic protrusions, a blind study was initially performed [20]. Each experiment was repeated at least three times using independent preparations.

### 2.14. Data Analysis

All data are expressed as the means ± SD. Statistical analyses were performed with SPSS Statistics for Windows, Version 20.0 (Armonk, NY, USA). Differences between the experimental groups were compared using one-way analysis of variance (ANOVA), and Student's* t*-test was used for comparisons. *p* < 0.05 was considered statistically significant.

## 3. Results

### 3.1. Expression of miR-132 and p250GAP Is Associated with Synaptogenesis and Electrophysiological Activity

**Figure 1:**
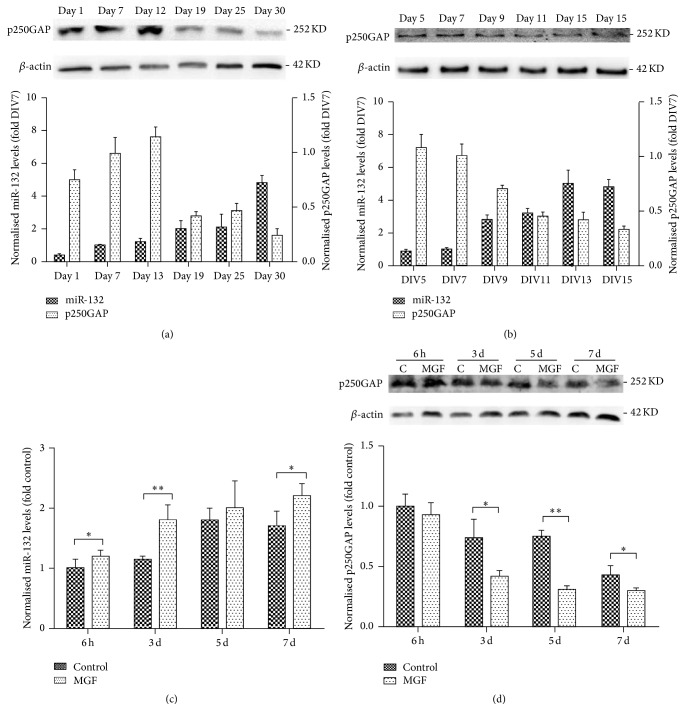
(a) The expression levels of miR-132 and p250GAP in C57BL/6 mice during postnatal d 1 to d 30 were evaluated using quantitative reverse transcription PCR and Western blot analyses. (b) The expression levels of miR-132 and p250GAP protein during DIV5–DIV15 in cultured hippocampal neurons. (c, d) Expression level of miR-132 and p250GAP 6 h to 7 d after MGF treatment as compared to control group. The data are presented as the mean ± SD values and subjected to ANOVA and Tukey's posttest. ^*∗*^ *p* < 0.05; ^*∗∗*^ *p* < 0.01 (*n* = 5; each data point represents mean ± SD of 5 experiments).

It has been reported that the expression level of mature miR-132 is low during the first week in the neonatal rat hippocampus, with a significant increase in miR-132 levels during weeks 2–4, which is also a critical period for the development and maturation of spines in rodents [15]. In our study, the expression levels of miR-132 and p250GAP in C57BL/6 mice during postnatal d 1 to d 30 were similar to those of previous studies (Figure 1(a)). We evaluated the chronological expression level of miR-132 and p250GAP during the maturation process of cultured hippocampal neurons. The level of miR-132 increased from DIV5 to DIV13 and was maintained at a relatively high level, whereas the expression of p250GAP protein was high at DIV5 but decreased at DIV15 (Figure 1(b)). This result indicated that the expression of miR-132 and p250GAP might correlate with the process of physiological spine maturation and synaptogenesis.

The expression levels of miR-132 and p250GAP at 6 h, 3 d, 5 d, and 7 d after MGF treatment were evaluated to determine whether the levels of miR-132 and p250GAP were influenced by the electrophysiological activity of cultured neurons. The results showed that miR-132 was upregulated 6 h to 7 d following magnesium-free medium treatment, with statistical significance at 6 h, 3 d, and 7 d, while p250GAP protein was downregulated from 6 h to 7 d following magnesium-free medium treatment, with statistical significance at 3 d, 5 d, and 7 d (Figures 1(c) and 1(d)).

The results suggest that miR-132 expression is increased during the active synaptogenesis periods of the immature brain. Moreover, the upregulation of miR-132 in the SRED model of hippocampal neurons suggests that the pathological electrical excitability may also influence miR-132 expression, which may correlate with the pathological synaptogenesis of the CNS during epileptogenesis.

### 3.2. miR-132 Regulates the Activation of Cdc42 via p250GAP in SRED Model of Hippocampal Neurons

**Figure 2:**
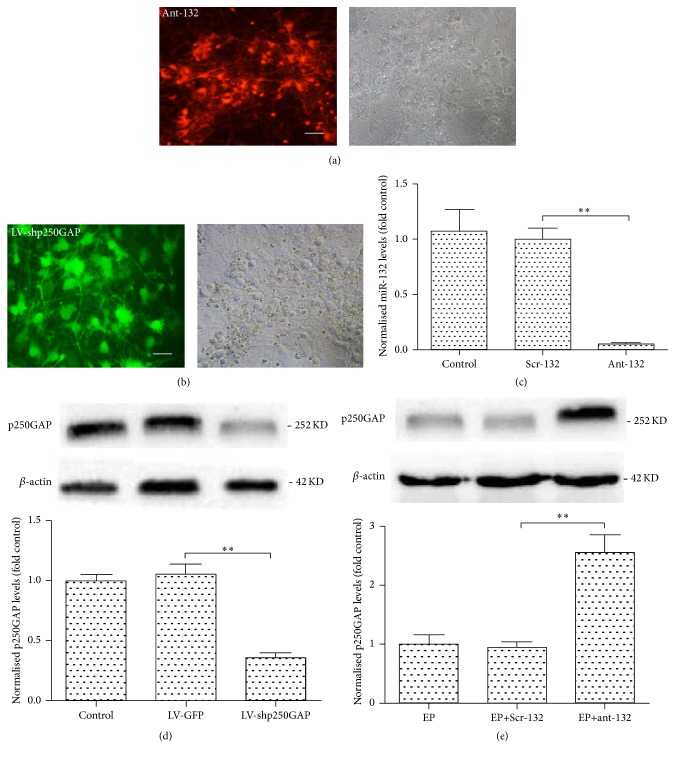
Knockdown of miR-132 and p250GAP in cultured neurons. (a) Cells were transfected with ant-132 at DIV7 and observed using a fluorescence microscope 48 h after transfection. Transfection efficiency was measured by RFP fluorescence. (b) Cells were transfected with LV-shp250GAP at DIV7 and observed 72 h after transfection. Transfection efficiency was measured by GFP fluorescence. Scale bar is 50 *µ*m. (c, d) Measurement of knocked down expression in transfected neurons was performed by qRT-PCR for miR-132 and by WB for p250GAP. (e) Expression level of p250GAP 72 h after ant-132 treatment in cultured epileptic hippocampal neurons. ^*∗∗*^ *p* < 0.01 (*n* = 10; the data are representative of 5 experiments).

We used a specific antagonist of miR-132 and RNA interference to knock down the expression of miR-132 and p250GAP (Figures 2(a) and 2(b)). The transfection efficiency was confirmed by qRT-CPR for miR-132 (Figure 2(c)) and by WB for p250GAP (Figure 2(d)). The expression level of p250GAP has significantly upregulated after ant-132 treatment which indicated that the expression level of p250GAP was negatively regulated by miR1-132 in our cultured epileptic hippocampal neurons (Figure 2(e)).

Rac1 and Cdc42 are considered two important promotors of dendritic branching and synaptic plasticity, while RhoA acts in the opposite manner [16]. p250GAP has been reported to mainly regulate the activation of Rac1 and Cdc42 in the CNS [16]. The pathological plasticity of neuronal spines and synapses is important in the pathological process of epilepsy; thus, we studied the activation levels of Rac1 and Cdc42 to explore whether Rac1 and Cdc42 are regulated by the miR-132/p250GAP pathway in our SRED model of cultured hippocampal neurons. The activation level of Rac1 and Cdc42 was tested.

**Figure 3:**
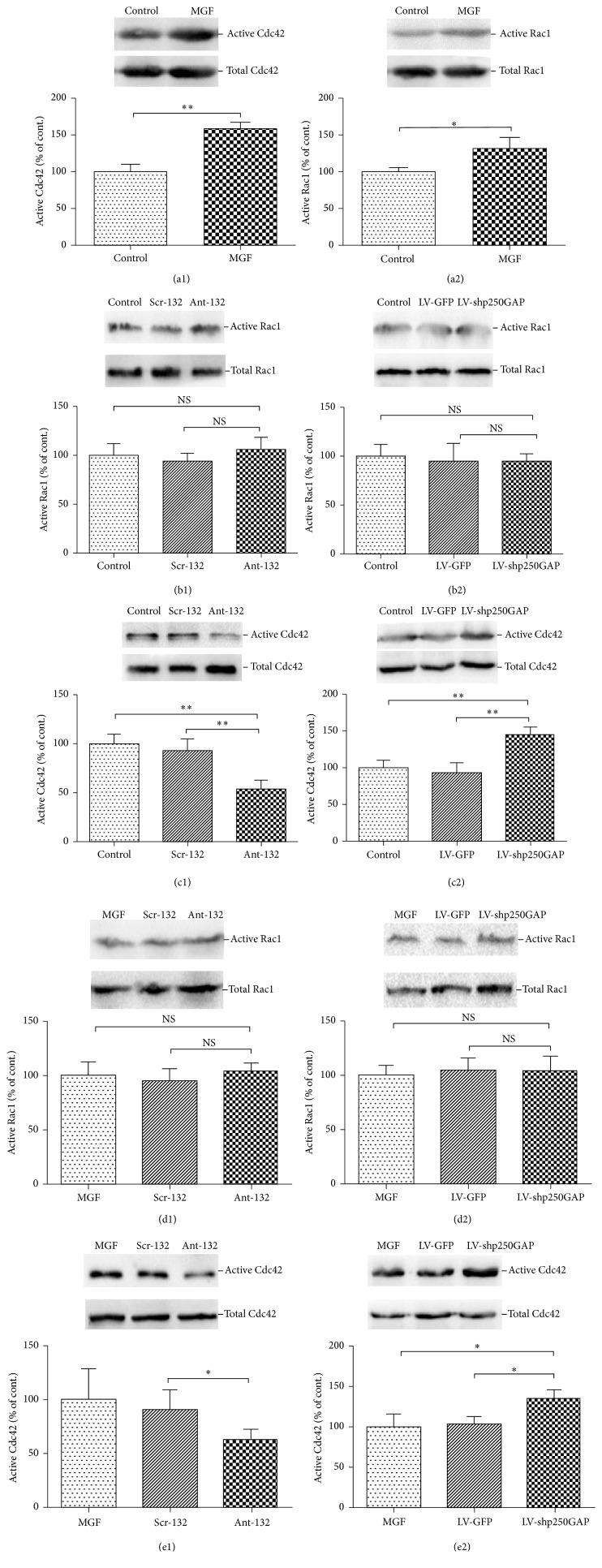
(a1-a2) The levels of active Cdc42 and Rac1 were significantly elevated in MGF-treated neurons compared to control neurons. (b1-b2) Effect of ant-132 and LV-shp250GAP transfection on active Rac1 levels. Cultured hippocampal neurons were transfected at DIV7 with ant-132 and LV-shp250GAP, and the level of active Rac1 was detected at DIV13. Transfection of ant-132 or LV-shp250GAP did not significantly affect Rac1 activity. (c1-c2) Cdc42 activation was significantly inhibited after ant-132 transfection and elevated after LV-shp250GAP transfection. (d1-d2) Effect of ant-132 and LV-shp250GAP transfection on Rac1 activation level in the MGF medium-treated hippocampal neurons. Cultured hippocampal neurons were transfected at DIV7 with ant-132 and LV-shp250GAP and then treated with MGF medium at DIV10. The level of active Rac1 was detected at DIV13. Rac1 activation level in the MGF medium-treated hippocampal neurons was not affected by ant-132 and LV-shp250GAP. (e1-e2) The activation level of Cdc42 in the MGF medium-treated hippocampal neurons was significantly affected by the inhibition of miR-132 and p250GAP expression. ^*∗*^ *p* < 0.05; ^*∗∗*^ *p* < 0.01 (*n* = 5; the data are representative of 5–7 experiments).

First, we found that the activation levels of both Rac1 and Cdc42 were significantly elevated in MGF-treated hippocampal neurons compared to control hippocampal neurons (Figures 3(a1) and 3(a2)). To determine whether and how Rac1 and Cdc42 are regulated by the miR-132/p250GAP pathway in our cultured hippocampal neurons, we evaluated the activation levels of Rac1 and Cdc42 in cultured hippocampal neurons when miR-132 or p250GAP expression was inhibited. The results showed that transfection of ant-132 or LV-shp250GAP did not significantly affect the activity of Rac1 (Figures 3(b1) and 3(b2)), while the activity of Cdc42 was significantly inhibited after ant-132 transfection and elevated after LV-shp250GAP transfection (Figures 3(c1) and 3(c2)). Furthermore, we obtained similar results in the MGF-treated SRED model of cultured hippocampal neurons, while the expression of miR-132 or p250GAP was inhibited (Figures 3(d1), 3(d2), 3(e1), and 3(e2)).

Our data suggest that p250GAP may primarily function as a GAP for Cdc42 in this epileptic model of cultured neurons.

### 3.3. miR-132 Influences Neuronal Electrical Excitability through p250GAP in SRED Model of Hippocampal Neurons

**Figure 4:**
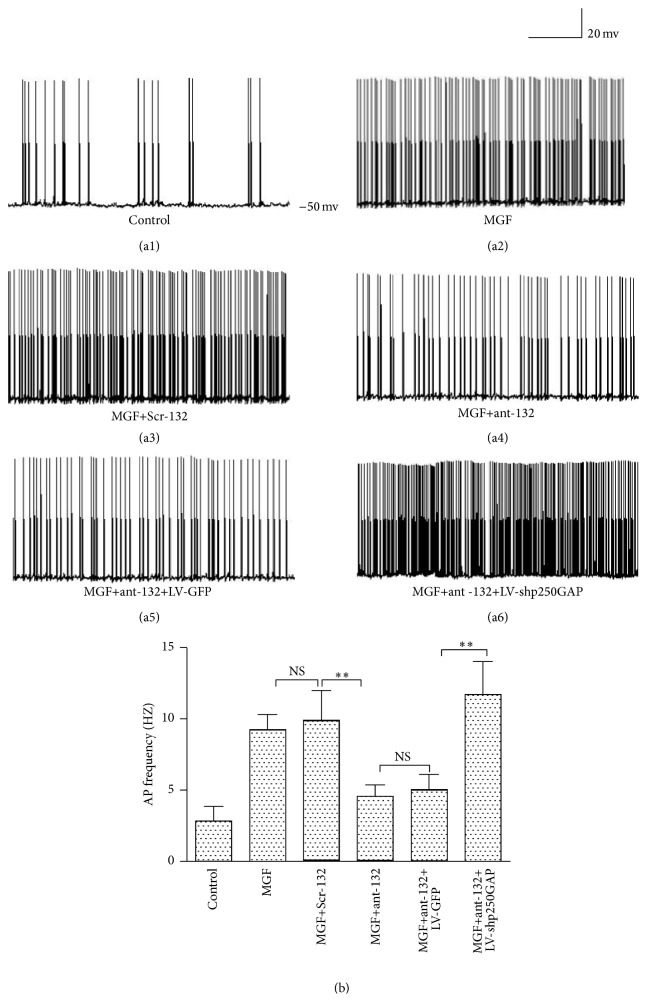
Representative trace of AP in cultured hippocampal neurons. Cultured hippocampal neurons were separately transfected at DIV7 with Scr-132, ant-132, ant-132+LV-GFP, and ant-132+LV-shp250GAP and then treated with MGF medium at DIV10. AP frequency was tested 24 h after the MGF treatment. (a1) Representative recordings of non-MGF medium-treated neurons (control). (a2) Representative recordings of MGF medium-treated neurons. (a3–a6) Representative recordings of MGF medium-treated neurons separately after Scr-132, ant-132, ant-132+LV-GFP, and ant-132+LV-shp250GAP transfection. (b) Statistical analysis of AP frequencies. ^NS^ *p* > 0.05, ^*∗*^ *p* < 0.05, and ^*∗∗*^ *p* < 0.01 (*n* = 24; the data are representative of 5 experiments).

To clarify the effect of miR-132 and p250GAP on neuronal excitability, we performed electrophysiological evaluations of the MGF medium-treated cultured hippocampal neurons. Our experiment showed that ant-132 treatment significantly decreased the AP frequency, suggesting that ant-132 can significantly inhibited neuronal electrical excitability in our SRED model of cultured hippocampal neurons (Figures 4(a2)–4(a4)). In contrast, ant-132 and LV-shp250GAP cotreatment significantly increased the AP frequency (Figures 4(a5) and 4(a6)).

### 3.4. Neuroprotective Effect of the miR-132/p250GAP Pathway on Cultured Neurons

**Figure 5:**
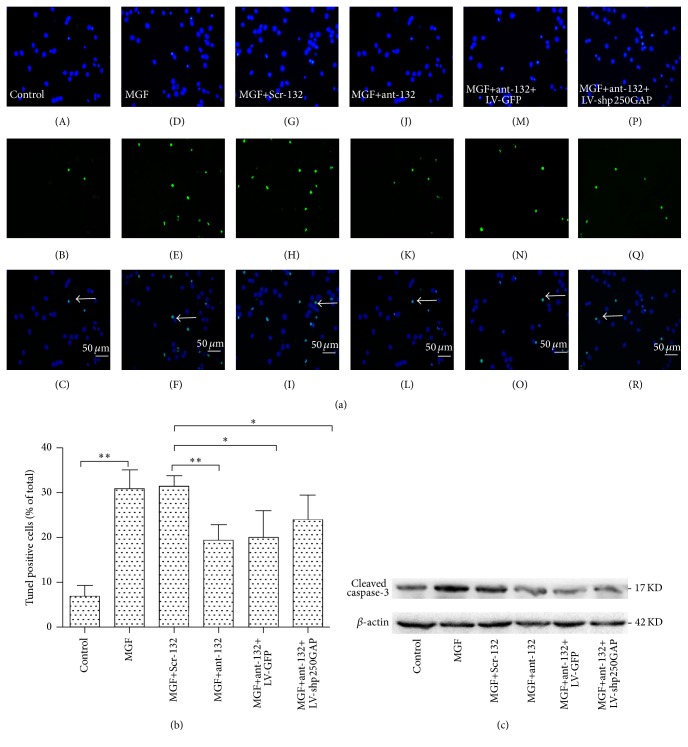
(a) Neuroprotective effect of ant-132 in cultured MGF-treated hippocampal neurons. TUNEL assay was used to evaluate the apoptosis level. Apoptotic cells in the non-MGF-treated control group (A–C). MGF medium-treated control group (D–F). Neurons pretreated with Scr-132 (G–I). Neurons pretreated with ant-132 (J–L). Neurons pretreated with ant-132+LV-GFP (M–O). Neurons pretreated with ant-132+LV-shp250GAP (P–R). The white arrow indicates the apoptotic cell. Scale bar is 50 *µ*m. (b) Histogram showing the percentage of cells with condensed nuclei in each group. The data are presented the mean ± SD values and subjected to ANOVA and Tukey's posttest. ^*∗*^ *p* < 0.05; ^*∗∗*^ *p* < 0.01 (*n* = 6; the data are representative of 5 experiments).

Silencing the overexpression of miR-132 is known to have a neuroprotective effect after acute brain injury [8, 20]; therefore, we asked whether miR-132 regulates neuronal apoptosis via the p250GAP pathway. We first transected the cultured neurons with ant-132 at DIV7 and then induced the SRED model at DIV10. Next, we silenced the expression levels of both miR-132 and p250GAP to investigate whether p250GAP was involved in the neuronal apoptosis effect of miR-132. TUNEL staining (Figures 5(a) and 5(b)) and WB detection of cleaved caspase-3 levels (Figure 5(c)) were performed 24 h later to investigate the level of apoptosis. Our results showed that regardless of whether the expression level of p250GAP was suppressed miR-132 silencing decreased neuronal apoptosis. This finding indicates that miR-132 silencing has a neuroprotective effect but that this effect may occur through pathways other than the p250GAP pathway.

### 3.5. miR-132 Silencing Decreases SRS in a Lithium-Pilocarpine Model* In Vivo*

**Figure 6:**
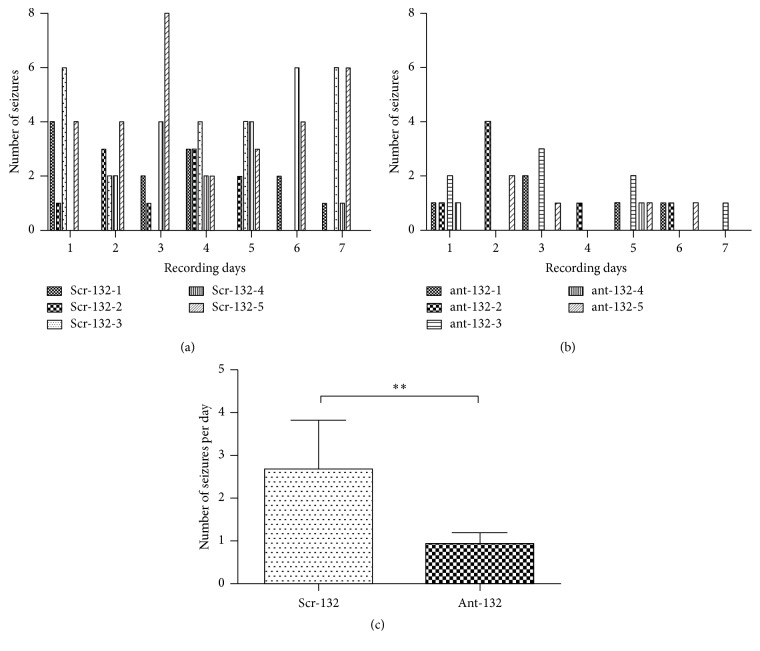
The number of recurrent spontaneous seizures in the epileptic chronic phase. (a) The number of spontaneous seizures per day for each mouse in the Scr-132 control group. (b) The number of seizures in the ant-132 group. (c) The mean number of epileptic seizures per day in the two groups. ^*∗∗*^ *p* < 0.01.

We then investigated whether miR-132 inhibition could suppress chronic seizures* in vivo* using a lithium-pilocarpine model. The mice were injected with ant-132 i.c.v. 48 h after SE. Continuous video monitoring was performed in the 6th week for 7 d. The frequency of chronic seizure was quantified and statistically analyzed by comparing the Scr-132 control group (Figure 6(a)) and the ant-132 group (Figure 6(b)). Statistical analysis of our behavioral investigation indicated that the ant-132 treatment can protect experimental mice against developing chronic recurrent seizures and can significantly decrease spontaneous seizure frequency (Figure 6(c)).

### 3.6. miR-132 Silencing Decreases the Remodeling of Spines in Mice with Chronic Seizures

**Figure 7:**
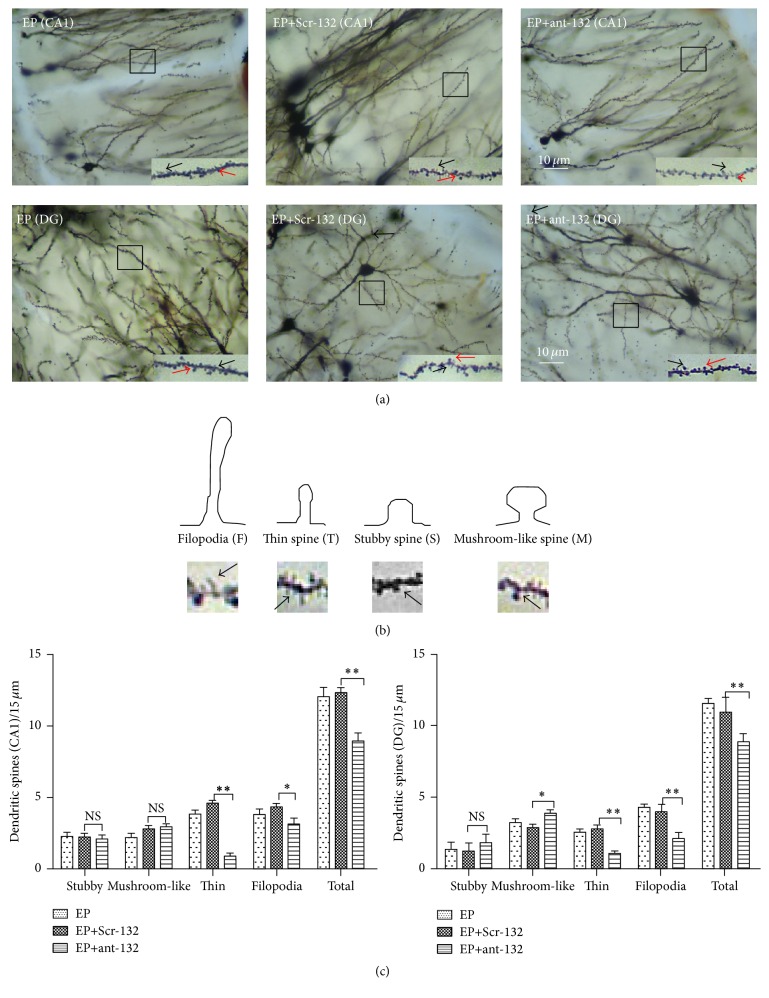
Golgi-Cox staining of hippocampal brain tissues for visualization of the dendritic spines. (a) Alteration of dendritic spines in the DG and CA1 region of hippocampal neurons visualized using Golgi staining. Scale bar is 10 *µ*m. The red arrows indicate the mushroom-like or stubby spines; black arrows indicate the filopodia or thin spines. (b) Schematic representation of spine classes. (c) Statistical analysis of the total number and different forms of spines between the EP group, EP+ant-132 group, and EP+Scr-132 group. ^NS^ *p* > 0.05, ^*∗*^ *p* < 0.05, and ^*∗∗*^ *p* < 0.01.

In general, mushroom-shaped and stubby spines represent more mature and stable spines, while thin spines and filopodia tend to be more plastic and immature (Figure 7(b)) [21]. Disrupted maintenance of the dendritic tree and spinogenesis are two important neurophysiological features of epilepsy. After the behavioral observations were completed, all the brain tissues of the ant-132-treated (EP+Ant-132), Scr-132-treated (EP+Scr-132), and nonintervention control (EP) groups were excised and Golgi-stained to further investigate the morphological effects of ant-132 treatment on the dendritic spine (Figure 7(a)). The total number and different forms of spines in the hippocampal dentate gyrus (DG) and CA1 regions were quantified and statistically analyzed to investigate the differences in spine density and morphology in the hippocampus of the experimental mice. We found that ant-132 treatment could decrease the spine density and elevate the proportion of stable spines (Figure 7(c)), indicating that the ant-132 treatment might help to suppress spine remodeling under the pathological condition of epilepsy.

## 4. Discussion

Four major findings were obtained in this study. First, the altered expression of miR-132 and p250GAP* in vivo* and* in vitro* indicated that miR-132-mediated p250GAP activity might be related to the remodeling process of spines. Second, miR-132/p250GAP regulates the activity of Cdc42 in a hippocampal neuronal culture model of acquired epilepsy, which is possibly the reason for dendritic spine remodeling during epileptogenesis because Cdc42 is an important cytoskeletal regulator that has been reported to trigger the outgrowth of peripheral spike-like protrusions called filopodia [22]. Third, ant-132 can decrease neuronal electrical excitability* in vitro* through p250GAP, and ant-132 can decrease chronic recurrent seizure development* in vivo*. Finally, the neuroprotective effect of ant-132 was once again verified in our experimental model of cultured neurons, and we revealed that this effect does not occur through the p250GAP pathway.

miR-132 is an activity-dependent gene that is implicated in synapse formation through regulating the translation of its target, p250GAP [2]. Physiological synapse formation in the immature brain and/or a bicuculline-induced increased in synaptic activity can change the expression levels of miR-132 and p250GAP. Conversely, altered expression of miR-132 and p250GAP can significantly affect dendritic spine density and head size [2]. Epilepsy is a pathological process of synaptic plasticity and excitatory loop formation that follows many types of acute brain injury and that can change neuronal activity [14, 15]. We proposed that miR-132-regulated p250GAP also participates in the pathological process of epilepsy. Although the upregulation of miR-132 has been observed in several epileptic models* in vivo* [2–5], no study has clarified the molecular mechanism of miR-132 in the epileptic process.

We have demonstrated the role of miR-132 and its target, p250GAP, in an epileptic model of cultured hippocampal neurons. The p250GAP protein can regulate Rho GTPase family members, including RhoA, Rac1, and Cdc42, and mediates actin cytoskeleton organization. Rac1/Cdc42 promote dendritic branching and growth; previous studies performed* in vivo* had inconsistent results about the ability of p250GAP to regulate the GTP-loaded state of Rac1 and Cdc42 [16, 22]. Here, we investigated the activation levels of the dendritic growth promoting factors Rac1/Cdc42 and further demonstrated, that under the condition of aberrant neuronal activity of epileptic discharge, miR-132 and p250GAP principally regulated the activation level of Cdc42, which indicated that Cdc42 might be a more appropriate target for further study about miR-132 and epilepsy. Although silencing miR-132 or p250GAP has no significant effect on the activation level of Rac1, the activation level of Rac1 has also upregulated in our cultured epileptic hippocampal neurons; further studies are needed to reveal if Rac1 has taken part in the epileptogenesis process.

Several studies have shown that miR-132 may regulate a significant increase in the density of spines and length of dendrites under the condition of aberrant neuronal activity* in vivo* [2, 15]. In our study, we attempted to detect the effect of miR-132 on spinous remodeling during epileptogenesis through morphological analysis of the dendritic spines of ant-132-treated pilocarpine-induced chronic epileptic mice. Treatment with ant-132 significantly increased the proportion of mushroom-shaped dendritic spines in the DG and CA1 regions of the hippocampus, which are two critical areas of pathological synaptic formation that leads to epilepsy. Because mushroom-shaped stubby dendritic spines are considered more mature and stable, while dendritic spines with other shapes, such as filopodia and thin spines, are immature and more plastic [23, 24], our experimental results indicate that ant-132 could be helpful for stabilize and maintaining the proper functioning of the dendritic spines of the hippocampus in the epileptogenesis process.

Treatment with ant-132 can decrease SRS* in vivo*. Our electrophysiological study of cultured neurons also confirmed that ant-132 can inhibit the AP frequency of magnesium-free medium-treated neurons and that this effect can be reversed if p250GAP is silenced. These results suggest that ant-132 may regulate neuronal electrical excitation, which is an important pathological change during epileptic formation, via the p250GAP pathway.

Several studies have reported that miR-132 overexpression can inhibit cell proliferation and promote cellular apoptosis [8, 21, 25]; however, the underlying molecular mechanism is still not clear. Our study verified that miR-132 upregulation promotes neuronal apoptosis in an epileptic model of cultured hippocampal neurons and that the neuroprotective effect of ant-132 still occurs even if p250GAP has been inhibited, thus indicating that other pathways are involved in the neuroprotective effect of ant-132.

## 5. Conclusion

In summary, we found that the expression of miR-132 and its target, p250GAP, are relevant to epileptogenesis. Silencing miR-132 can decrease chronic recurrent seizures. miR-132 likely functions through regulating the expression of p250GAP, the activation of downstream Cdc42, and the maintenance of the proper form and function of the dendritic spines, which may affect neural electrical activity of neurons and epileptogenesis. Treatment with ant-132 has a neuroprotective effect; however, this effect is not mediated through the p250GAP pathway. Further research is needed to reveal the detailed mechanism.
